# Neuroprotective activity of a virus‐safe nanofiltered human platelet lysate depleted of extracellular vesicles in Parkinson's disease and traumatic brain injury models

**DOI:** 10.1002/btm2.10360

**Published:** 2022-07-12

**Authors:** Liling Delila, Ouada Nebie, Nhi Thao Ngoc Le, Lassina Barro, Ming‐Li Chou, Yu‐Wen Wu, Naoto Watanabe, Masayasu Takahara, Luc Buée, David Blum, David Devos, Thierry Burnouf

**Affiliations:** ^1^ Graduate Institute of Biomedical Materials and Tissue Engineering, College of Biomedical Engineering Taipei Medical University Taipei Taiwan; ^2^ Univ. Lille, Inserm, CHU‐Lille, U1172, Lille Neuroscience & Cognition Lille France; ^3^ Alzheimer & Tauopathies Labex DISTALZ Lille France; ^4^ International PhD Program in Biomedical Engineering, College of Biomedical Engineering Taipei Medical University Taipei Taiwan; ^5^ Asahi Kasei Medical Co., Ltd. Tokyo Japan; ^6^ NeuroTMULille Lille Neuroscience & Cognition Lille France; ^7^ NeuroTMULille Taipei Medical University Taipei Taiwan; ^8^ International PhD Program in Cell Therapy and Regeneration Medicine Taipei Medical University Taipei Taiwan; ^9^ PhD Program in Graduate Institute of Mind Brain and Consciousness, College of Humanities and Social Sciences Taipei Medical University Taipei Taiwan; ^10^ Neuroscience Research Center Taipei Medical University Taipei Taiwan; ^11^ Present address: National Center of Blood Transfusion Ouagadougou Burkina Faso; ^12^ Present address: Institute of Clinical Medicine, National Yang Ming Chiao Tung University Taipei Taiwan

**Keywords:** human platelet lysate, nanofiltration, neuroprotection, prion, virus

## Abstract

Brain administration of human platelet lysates (HPL) is a potential emerging biotherapy of neurodegenerative and traumatic diseases of the central nervous system. HPLs being prepared from pooled platelet concentrates, thereby increasing viral risks, manufacturing processes should incorporate robust virus‐reduction treatments. We evaluated a 19 ± 2‐nm virus removal nanofiltration process using hydrophilic regenerated cellulose hollow fibers on the properties of a neuroprotective heat‐treated HPL (HPPL). Spiking experiments demonstrated >5.30 log removal of 20–22‐nm non‐enveloped minute virus of mice‐mock particles using an immuno‐quantitative polymerase chain reaction assay. The nanofiltered HPPL (NHPPL) contained a range of neurotrophic factors like HPPL. There was >2 log removal of extracellular vesicles (EVs), associated with decreased expression of pro‐thrombogenic phosphatidylserine and procoagulant activity. LC‐MS/MS proteomics showed that ca. 80% of HPPL proteins, including neurotrophins, cytokines, and antioxidants, were still found in NHPPL, whereas proteins associated with some infections and cancer‐associated pathways, pro‐coagulation and EVs, were removed. NHPPL maintained intact neuroprotective activity in Lund human mesencephalic dopaminergic neuron model of Parkinson's disease (PD), stimulated the differentiation of SH‐SY5Y neuronal cells and showed preserved anti‐inflammatory function upon intranasal administration in a mouse model of traumatic brain injury (TBI). Therefore, nanofiltration of HPL is feasible, lowers the viral, prothrombotic and procoagulant risks, and preserves the neuroprotective and anti‐inflammatory properties in neuronal pre‐clinical models of PD and TBI.

**Abbreviations**
BCA: bicinchoninic acid
CCI: controlled cortical impact
DENV: dengue virus
DLS: dynamic light scattering
DMEM: dulbecco's modified eagle medium
EGF: epidermal growth factor
EV: extracellular vesicle
FBS: fetal bovine serum
GO: gene ontology
HBV: hepatitis B virus
HCV: hepatitis C virus
HIV: human immunodeficiency virus
HPL: human platelet lysate
HPPL: heat‐treated human platelet pellet lysate
i.n: intranasal
KEGG: Kyoto encyclopedia of genes and genomes
LC‐MS: liquid chromatography‐mass spectrometry
LRV: log reduction value
MVM‐MVP: minute virus of mice‐mock virus particle
NHPPL: nanofiltered heat‐treated human platelet pellet lysate
NTA: nanoparticle tracking analysis
PBS: phosphate buffered‐saline
PC: platelet concentrate
PDGF‐AB: platelet‐derived growth factor‐AB
PEV: platelet extracellular vesicle
PS: phosphatidylserine
RA: retinoic acid
VEGF: vascular endothelial growth factor
WNV: West Nile virus
ZIKV: Zika virus

## 1 INTRODUCTION

No efficient pharmacological treatment to balance the multifaceted cognitive and motor functional deterioration associated with neurodegeneration and brain trauma is developed.^1^, ^2^, ^3^ Such relative failures contrast with substantial therapeutic achievements made in treating other pathologies. Therefore, a safe, practical, accessible, and affordable therapy of brain pathologies is still needed. Accumulating preclinical data now suggest that the human platelet lysate (HPL) proteome, made of a physiological combination of trophic factors, could emerge as a novel multifaceted biotherapy to treat diseases affecting the central nervous system (CNS).^4^, ^5^, ^6^, ^7^ Intense neuroprotective actions of HPL administration to the brain by intracranial/cerebroventricular or intranasal (i.n.) routes are found in animals models of stroke,^8^ Parkinson's disease (PD),^9^, ^10^ Alzheimer's disease (AD),^11^, ^12^ traumatic brain injury (TBI),^13^ or amyotrophic lateral sclerosis (ALS).^14^ These in vivo results confirmed the convincing protective effects previously observed in cellular models of neurological diseases.^15^, ^16^ Most studies have linked the neuroprotective benefits of HPLs to their unique physiological mix of neurotrophic growth factors, cytokines, antioxidants, anti‐inflammatory molecules, and neurotransmitters, present in a soluble form or possibly loaded in extracellular vesicles (EVs).^17^, ^18^, ^19^, ^20^, ^21^ The complex platelet proteome can synergistically activate complementary protective biological pathways and counterbalance pathological gene and protein expressions resulting from brain disorders and trauma.^9^, ^12^, ^13^ For example, in vitro and in vivo studies showed that platelet lysate treatment can (a) protect against progressive or acute loss of synapses, (b) restore neuronal integrity, and (c) counterbalance neuroinflammation, oxidative stress, and defects in cognitive and/or motor functions.^4^, ^5^, ^6^, ^15^, ^22^

Translational applications of HPL made from pooling platelet concentrates (PCs) from blood donors are now being considered, with clinical trials planned in patients with ALS.^14^, ^23^ HPLs for brain administration should have optimal quality and safety and meet established specifications. Safety requirements led us to develop a dedicated HPL, termed HPPL (for heat‐treated human platelet pellet lysate), that is depleted of plasma, of relatively low protein content to avoid overloading the cerebrospinal fluid, essentially free of neurotoxic or clottable proteins, and with low pro‐thrombogenic, proteolytic, and proinflammatory activities.^10^, ^13^ As HPPL is made from human blood, a vital concern is avoiding the risk of transfusion‐transmitted infections. Virus safety is particularly critical for HPLs manufactured from multiple allogeneic PCs to ensure quality consistency since pooling increases contamination risks.^24^, ^25^, ^26^ It is crucial to prevent contamination by highly pathogenic blood‐borne viruses like human immunodeficiency virus (HIV) and hepatitis B virus (HBV) and various blood‐borne viruses exerting brain neurotoxicity such as hepatitis C virus (HCV), Dengue virus (DENV), Zika virus (ZIKV), West Nile virus (WNV), or, potentially, Human simplex virus (HSV) or severe acute respiratory syndrome coronavirus (SARS‐CoV)‐2 (which does not seem transmissible by blood).^27^, ^28^ Preventative measures include careful screening of healthy candidate blood donors and viral testing of individual donations by serological and nucleic acid tests.^29^ However, the optimal viral safety margin requires implementing dedicated robust virus‐inactivation or ‐removal treatments that do not affect the products' therapeutic safety and efficacy,^28^, ^30^, ^31^ and can be applied to pooled platelet biomaterials.^24^, ^25^

**FIGURE 1:**
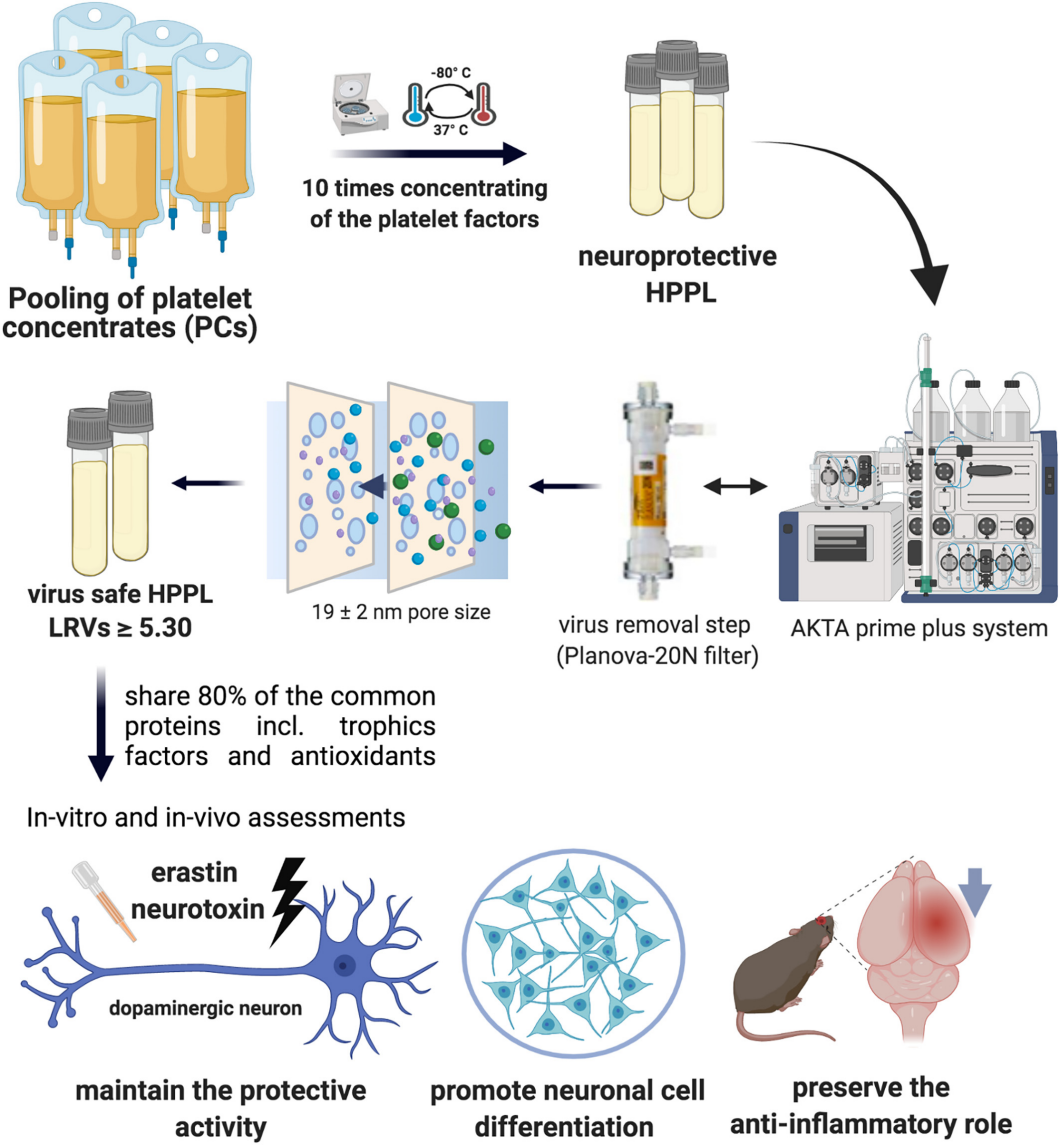
Overall experimental design.

We hypothesized that one virus‐reduction technology ideally suited for HPPLs should be “nanofiltration". Nanofiltration is a bioprocessing procedure of virus removal used in the plasma product industry;^30^, ^31^ a purified protein solution is filtered through a device made of multiple, cuprammonium‐regenerated cellulose hollow fibers, which have a cutoff of a few nanometers that is small enough to retain viruses, but large enough to let proteins flow through the nanosized membranes. To ensure a good compromise between the risks of clogging the filter by large proteins and virus‐removal efficiency, we thought that a nanofilter with a cutoff of 19 ± 2 nm should remove, by membrane entrapment, blood‐borne viruses, including the ca. 35–45‐nm neurotoxic flaviviruses (HCV, DENV, ZIKV, and WNV) and coronaviruses, as well as the ca. 150‐nm HSV.^30^, ^31^ However, we were uncertain as to how this virus‐removal step would affect the HPPL proteome and EV content and, as a result, its neuroprotective activities. To answer these questions, we first developed conditions to nanofilter HPPL and then verified, by spiking experiments using 20–22‐nm minute virus of mice‐mock virus particles (MVM‐MVPs), the extent of virus removal by an immuno‐quantitative polymerase chain reaction (qPCR) assay. We then characterized the impact of this nanofiltration step on the HPPL composition, including neurotrophic factors, EVs content, prothrombotic and procoagulant qualities, using various proteomic and biophysical assays. Finally, we used validated cellular and in vivo models of PD and TBI to assess the neuroprotective and anti‐inflammatory functions of the nanofiltered HPPL (NHPPL). This experimental design is illustrated in Figure 1.

## 2 RESULTS

### 2.1 Characterization of starting PCs

Pooled PC donations had a mean platelet number of 587 × 10^3^ cells/mm^3^. Residual red blood cells (RBCs at <0.7 × 10^6^ cells/mm^3^) and white blood cells (WBCs at <0.8 × 10^3^ cells/mm^3^) were undetectable, meeting the standard specifications for clinical applications in transfusion and our requirements for preparing HPPL.

### 2.2 HPPL nanofiltration

#### 2.2.1 Feasibility and virus‐removal experiments

HPPL made from pooled PCs following our established methods^10^, ^13^ was pre‐filtered using 0.2‐ and 0.1‐μm filters. We could readily filter close to 20 ml of this HPPL through 0.001 m^2^ Planova‐20N within approximately 3 h at a constant flow‐rate of 0.1 ml/min and without reaching the maximum pressure of 0.098 MPa fixed by the supplier. The transmembrane pressure recorded during Planova 20N filtration is shown in Figure S1. Therefore, the nanofiltration of the HPPL on Planova 20N was achieved without clogging. The capacity of a specially manufactured Planova‐20N with a filtration area of 0.0001 m^2^ to effectively remove small viruses was assessed by spiking MVPs into 4 ml of pre‐filtered 0.2–0.1‐μm HPPL, to reach an expected final concentration of 10^10^ MVPs/ml. MVP concentrations in spiked HPPL were determined by immuno‐qPCR to be 1.63 × 10^9^ ± 8.2 × 10^8^ and 1.83 × 10^9^ ± 1.77 × 10^8^, respectively, when immediately frozen after spiking or after being kept at room temperature during the duration of the nanofiltration experiment. This indicated no loss in detectable MVM associated with spiking into HPPL or storage at room temperature followed by freezing, demonstrating the absence of detrimental interference of the test material and the processing steps of the immuno‐qPCR assay. Ct values obtained by immuno‐qPCR in the NHPPL were the same as the baseline value of the unspiked control, thereby confirming absence of MVP, indicating a log reduction value (LRV) ≥5.39 log by nanofiltration (Table 1).

**TABLE 1:** Virus removal capacity

Volume spiked	Volume collected	Pressure	Duration
4 ml	1.83 ± 0.11 ml	0.08 MPa	30 min

MVP spiked value/ml	Spiked MVP value/ml	Log reduction value (LRV)
1 × 10^10^	(a) 1.63 × 10^9^ ± 8.2 × 10^8^	≥5.39
	(b) 1.73 × 10^9^ ± 4.97 × 10^8^	≥6.15

*Note*: (a) Immediately frozen at −80°C after spiking; (b) frozen at −80°C after being kept at room temperature during the nanofiltration duration. *N* = 2 for each condition.

Abbreviation: MVP, mock virus particle.

### 2.3 Characterization of NHPPL

#### 2.3.1 Total proteins and contents of trophic factors

We then decided to study impacts of nanofiltration on protein content and three selected growth factors. The total protein content decreased from ca. 7 mg/ml in HPPL to ca. 4 mg/ml after 0.2–0.1‐μm filtration, and ca. 3 mg/ml after Planova 20N. The respective content (ng/ml) in platelet‐derived growth factor (PDGF)‐AB, epidermal growth factor (EGF), and vascular endothelial growth factor (VEGF) in HPPL, 0.2–0.1‐μm‐filtered HPPL, and NHPPL was: 89.28 ± 9.67, 60.86 ± 11.84, 20.54 ± 3.05 (PDGF); 6.24 ± 0.75, 4.75 ± 0.42, 5.30 ± 0.36 (EGF); and 1.17 ± 0.02, 0.97 ± 0.03, 0.7 ± 0.01 (VEGF).

#### 2.3.2 Platelet extracellular contents and related procoagulant functional activities

**FIGURE 2:**
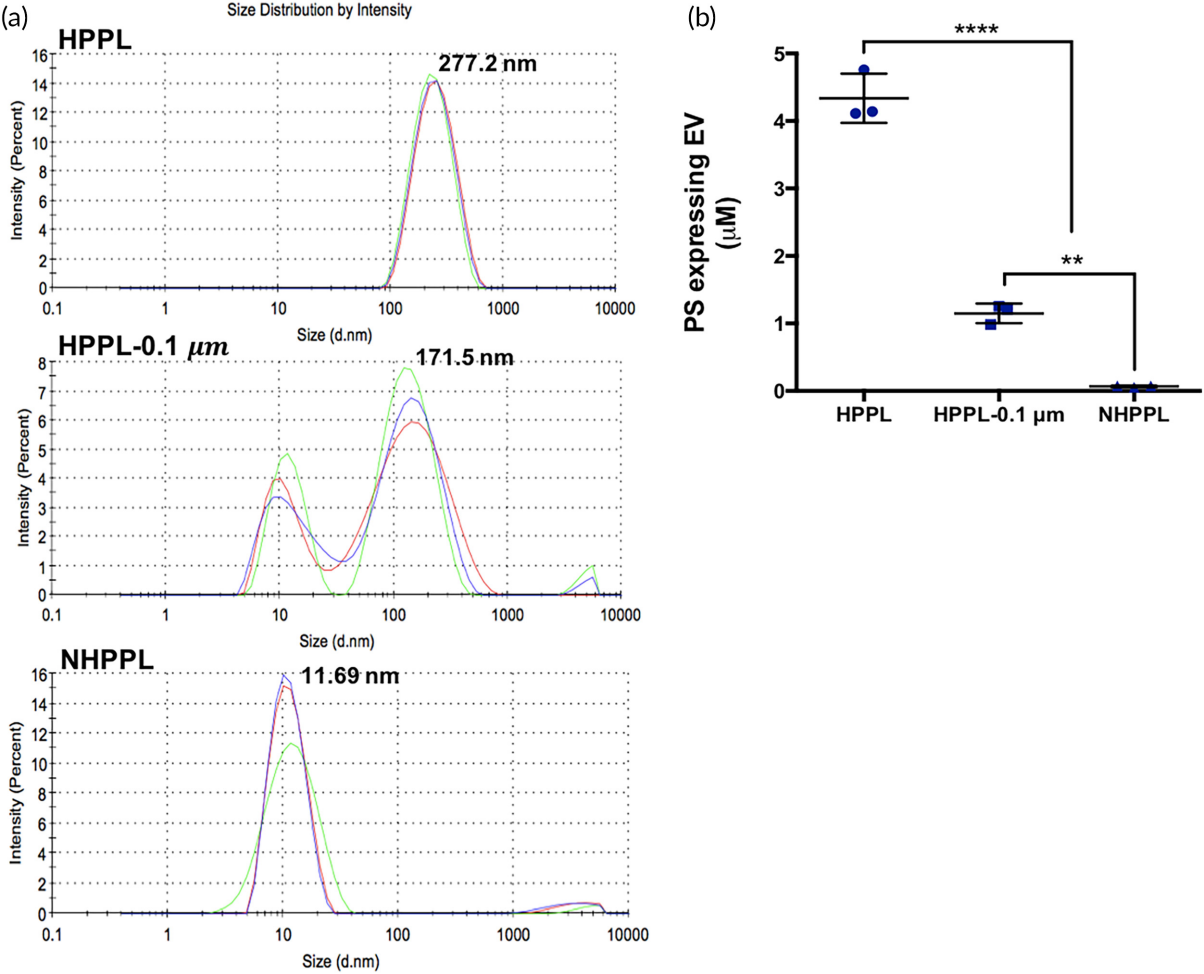
Platelet extracellular vesicle (PEV) content of heat‐treated platelet pellet lysate (HPPL), after 0.2–0.1‐μm filtration (HPPL‐0.1 μm), and of nanofiltered HPPL (NHPPL). (a) PEV size distribution analysis by dynamic light scattering. The red, green, and blue curves represent the first, second, and third analyses, respectively. (b) Phosphatidylserine‐expressing PEVs. Results are expressed as the means ± SD (*n* = 3). (**p < 0.01, ****p < 0.0001), compared to HPPL. Statistical evaluation was performed by a one‐way ANOVA followed by Fisher's least significant difference test.

We next assessed the size distribution and number of platelet‐EVs (PEVs) in HPPL before and after nanofiltration using dynamic light scattering (DLS) and nanoparticle tracking analysis (NTA). DLS evidenced a significant lowering of the PEV mean size distribution, which decreased from ca. 171.5 nm to ca. 10.5 nm after nanofiltration (Figure 2a), and the NTA showed ca. 90% reduction in the PEV concentration from 6.20 × 10^10^ ± 3.48 × 10^8^ to 6.21 × 10^9^ ± 3.12 × 10^8^ (Figure S2). Moreover, the STA‐procoagulant‐phospholipid assay, that specifically measures the impact of PEVs as a contributor to blood coagulation, showed a significant prolongation in the coagulation time by the NHPPL (of ca. 110 s) compared to the non‐nanofiltered HPPL (of ca. 24 s), consistent with removal of PEVs contributing to blood coagulation. Furthermore, the content of phosphatidylserine (PS)‐expressing EVs (Figure 2b) was significantly less in NHPPL compared to the crude (*p* < 0.0001) and the 0.2–0.1‐μm‐filtered (*p* < 0.01) HPPL. Thus, this nanofiltration process contributed to a substantial removal of EVs and to a significant decrease in its procoagulant effect.

#### 2.3.3 Proteomics analysis

**FIGURE 3:**
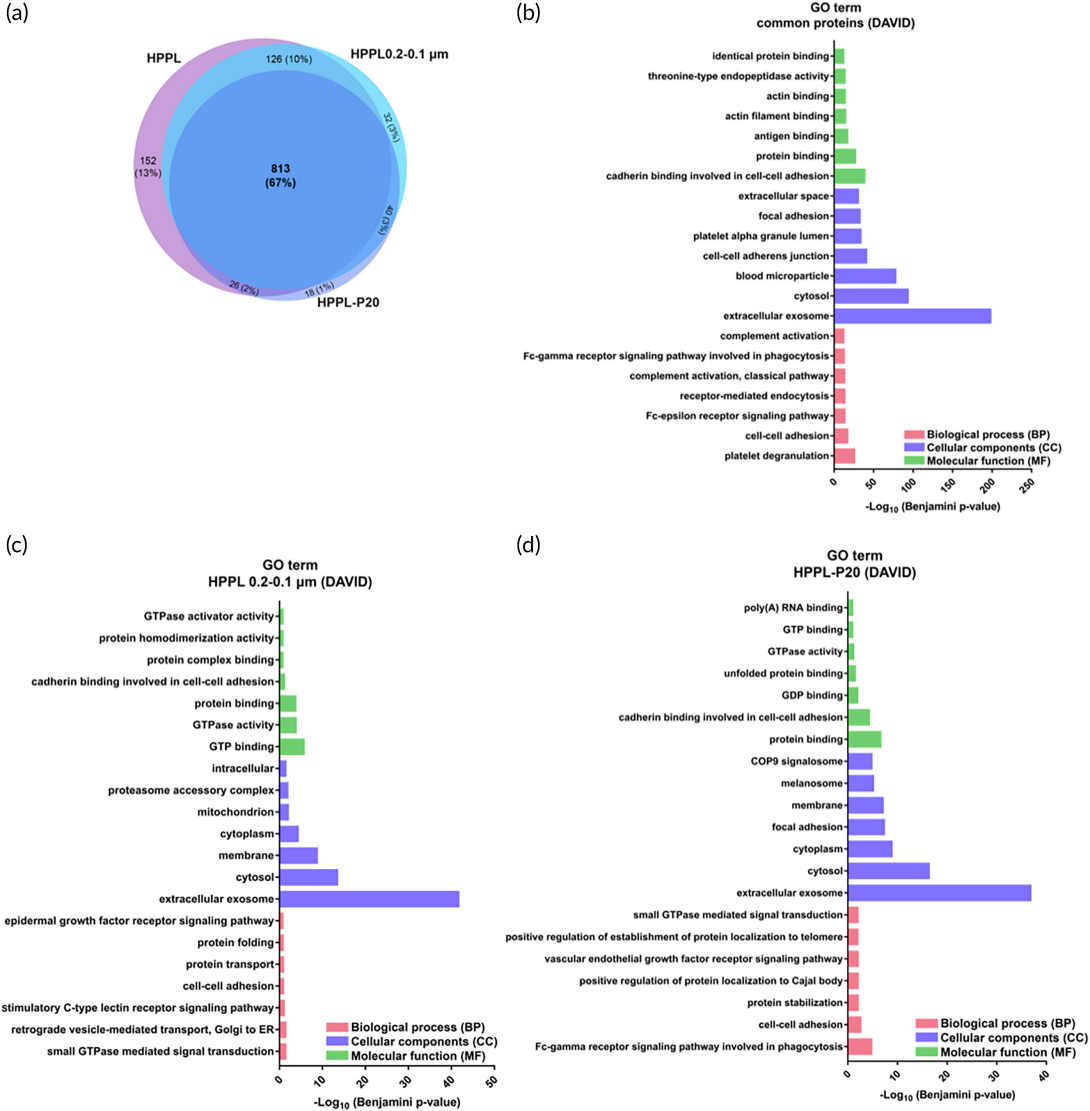
(a) Venn diagram of the proteins identified in heat‐treated platelet pellet lysate (HPPL), 0.2–0.1‐μm‐filtered HPPL (HPPL 0.2–0.1 μm), and nanofiltered HPPL (NHPPL) (HPPL‐P20). (b) Gene ontology enrichment analysis of 813 proteins in common among HPPL, HPPL 0.2–0.1 μm, and HPPL P‐20: biological processes (BPs), molecular functions (MFs), and cellular components (CCs). (c) Gene ontology enrichment analysis of proteins that were removed from HPPL by 0.2–0.1‐μm filtration and (d) removed from HPPL 0.2–0.1‐μm filtration by Planova 20N: BPs, MFs, and CCs. The significance cutoff was *p* < 0.1.

Totals of 1117, 1011, and 897 proteins (with a false discovery rate [FDR] of <1%, protein level FDR) were respectively identified in HPPL, 0.2–0.1‐μm‐filtered HPPL, and NHPPL (Figure 3a) by liquid chromatography‐tandem mass spectrometry (LC‐MS/MS). Eight hundred thirteen proteins were common across all samples. Interestingly, antioxidants (including superoxide dismutase, ceruloplasmin, glutathione peroxidase, gelsolin, and catalase), neurotrophic factors, chemokines, and cytokines (such as PDGF, VEGF, EGF, hepatocyte growth factor [HGF], glial maturation factor beta [GMFB], PF4, and CCL5) were all detectable in NHPPL (Table S2). Genes ontology (GO) analysis (Figure 3b) of the 813 common proteins revealed biological processes (BPs) associated with platelet degranulation, cell‐cell adhesion, the Fc‐epsilon receptor signaling pathway, receptor‐mediated endocytosis, the classical complement activation pathway, the Fc‐gamma receptor signaling pathway involved in phagocytosis, and complement activation. An analysis of cellular components (CC) showed that the top three subcategories involved extracellular exosomes, cytosol, and blood microparticles. The molecular function (MF) analysis revealed proteins mainly linked to cadherin binding associated with cell‐cell adhesion, protein binding, antigen binding, and actin filament binding.

One hundred fifty‐two proteins present in HPPL became undetectable after 0.2–0.1‐μm filtration, and an additional 158 proteins after Planova‐20N. The GO term analysis indicated that proteins removed, at least partially, by both 0.2–0.1‐μm filtration (Figure 3c) and then Planova‐20N (Figure 3d) were associated with extracellular exosomes and cytoplasm/cytosol. They act as binding proteins and exert various functions in cell adhesion and signaling pathways. Moreover, the abundances of GPIb, GPV, GVI—which are platelet EV membrane proteins expressed by non‐activated platelets, and CD‐62P/p‐selectin expressed by activated platelets, decreased after filtration.

**FIGURE 4:**
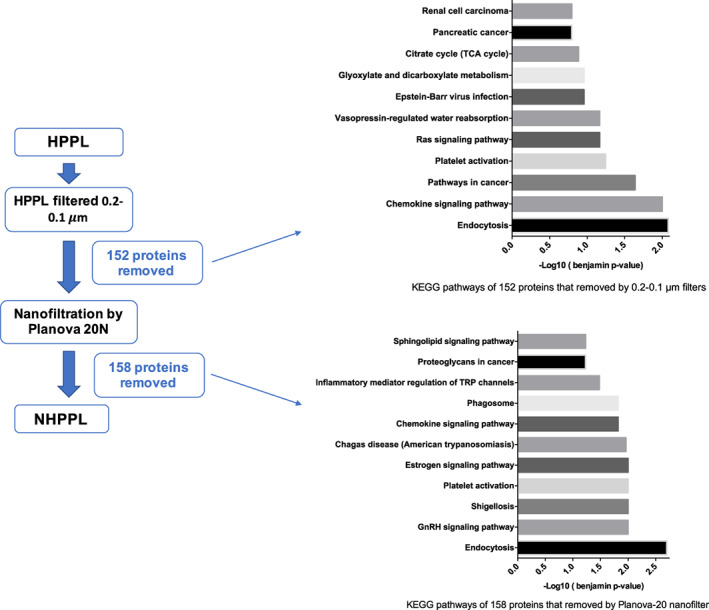
Kyoto encyclopedia of genes and genomes pathways of 158 proteins that were removed by 0.2–0.1‐μm filters and pathways of 152 removed by Planova‐20N filtration. The significance cutoff was *p* < 0.1.

The Kyoto Encyclopedia of Genes and Genomes (KEGG) pathway enrichment analysis of common proteins performed using DAVID identified pathways for complement and coagulation cascades, platelet activation, and the proteasome, with decreased abundances after both 0.2–0.1‐μm filtration and Planova‐20N (Figure S3). The KEGG pathway analysis performed for proteins removed by the two filtration processes showed platelet activation, endocytosis, and chemokine signaling‐related pathways among HPPL proteins but undetectable after 0.2–0.1‐μm filtration and Planova‐20N filtration (Figure 4). Interestingly, pathways related to diseases such as bacterial/viral/parasitic infections (e.g., Epstein–Barr virus infection and shigellosis) and some cancers (e.g., pancreatic cancer and renal cell carcinoma) were associated with proteins removed after 0.2–0.1‐μm filtration and such as shigellosis and Chagas disease after Planova‐20N filtration.

### 2.4 Functional activity of nanofiltered HPPL

#### 2.4.1 In vitro neuroprotective activity of NHPPL on Lund human mesencephalic cells exposed to the erastin neurotoxin

**FIGURE 5:**
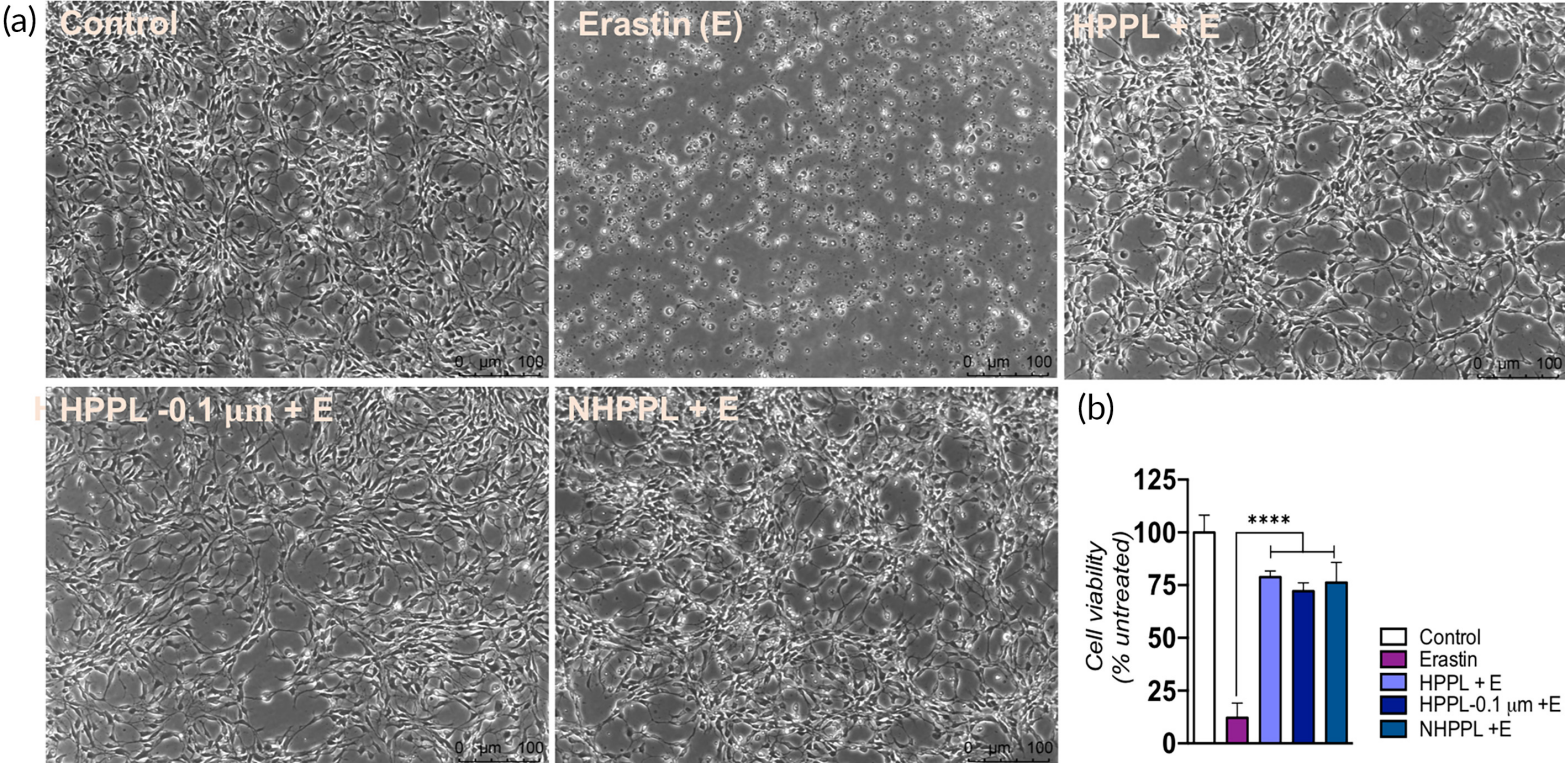
Assessment of the neuroprotective activity. LUHMES cells were pretreated with 5% (v/v) heat‐treated platelet pellet lysate (HPPL), 0.2–0.1‐μm‐filtered HPPL (HPPL‐0.1 μm), or nanofiltered HPPL (NHPPL) and after 1 h were exposed to the erastin neurotoxin. (a) Cell morphology after 24 h. The scale bar is 100 μm. (b) LUHMES cell viability after 24 h was quantified by a CCK‐8 assay. *n* = 3, ****p < 0.0001, compared to cells treated with erastin only. Statistical evaluation was performed by a one‐way ANOVA followed by Fisher's least significant difference test.

Differentiated Lund human mesencephalic (LUHMES) cells were treated with 2% (v/v) HPPL, 0.2–0.1‐μm‐filtered HPPL, and NHPPL for 1 h before exposure to 1 μM erastin, a neurotoxic agent that programs cell death via ferroptosis. Microscopic observations of LUHMES cells (Figure 5a) evidenced a normal morphology after treatment with HPPL and NHPPL, in contrast to untreated cells exposed to erastin only. CCK‐8 cell viability results, assessed after 24 h of erastin exposure, are shown in Figure 5b. NHPPL provided a significant neuroprotective effect against erastin neurotoxicity (*p* < 0.0001), similar to that achieved by HPPL and 0.2–0.1‐μm‐filtered HPPL (*p* < 0.0001). These results highlighted that nanofiltration did not affect the in vitro neuroprotective properties of HPPL.

#### 2.4.2 Ability of NHPPL to stimulate cell maturation in SH‐SY5Y

**FIGURE 6:**
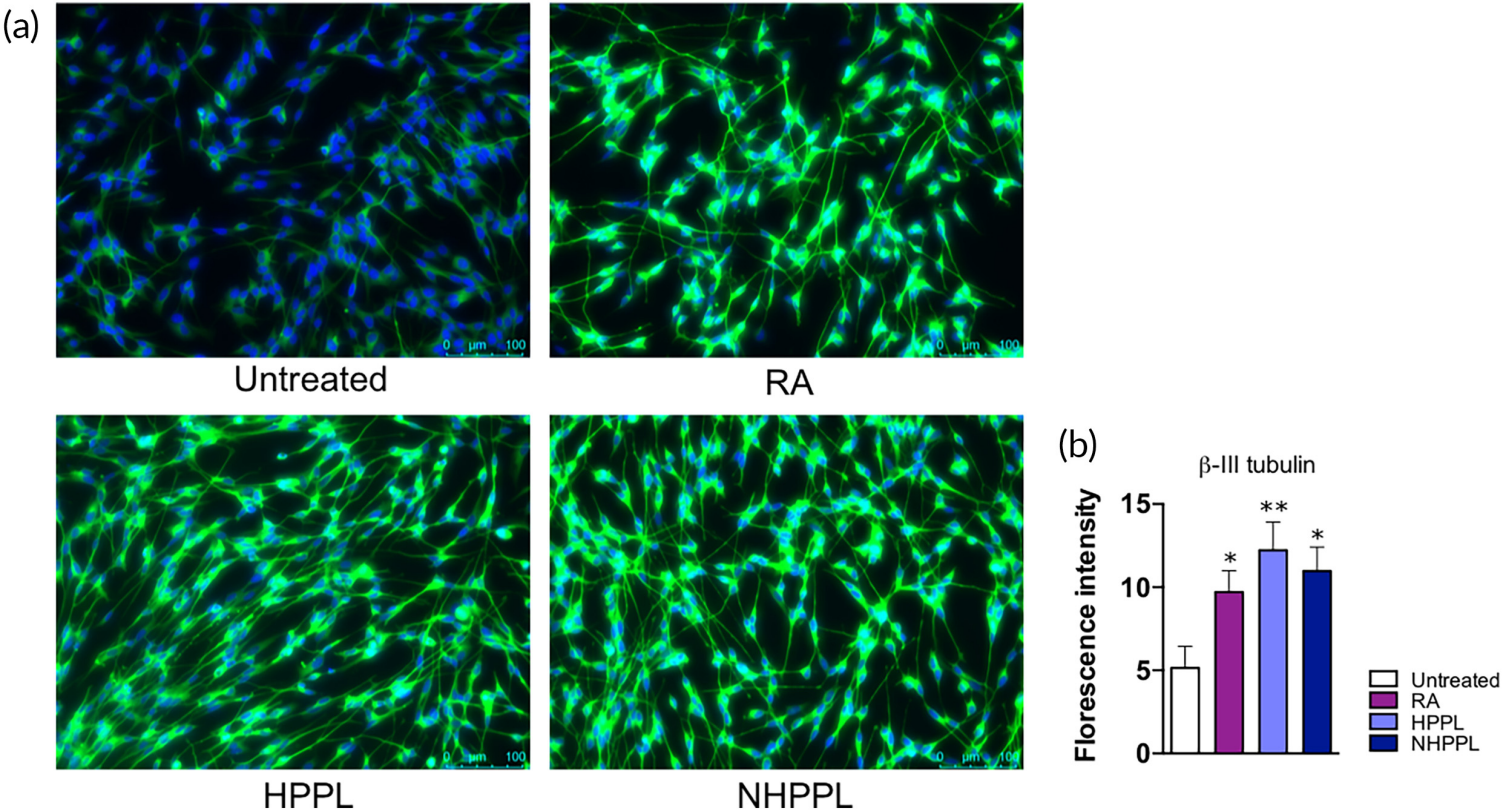
Capacity to stimulate SH‐SY5Y cell neuronal maturation. Cells were immuno‐stained with β‐III tubulin and counterstained with DAPI. Images (a) showing extension of SH‐SY5Y neurites under treatment with nanofiltered heat‐treated platelet pellet lysate (HPPL; NHPPL). HPPL, and retinoic acid which was used as a positive control to stimulate cell differentiation. The scale bar is 100 μm. (b) Quantitative measurement of the fluorescence intensity using ImageJ software. *p < 0.05; **p < 0.01. Statistical evaluation was performed by a one‐way ANOVA followed by Fisher's least significant difference test.

We determined the capacity of NHPPL to induce the neuronal differentiation of SH‐SY5Y cells in the absence of retinoic acid (RA), the usual neuronal differentiation agent. Undifferentiated SH‐SY5Y cells were grown in Dulbecco's Modified Eagle Medium (DMEM) supplemented with 10% fetal bovine serum (FBS), then, on day 2, the medium was changed to DMEM only, without FBS, and cells were treated by 2% (v/v) NHPPL, or with 2% (v/v) of HPPL or 1 μM RA (positive controls). Some cells were maintained using a medium with 0.5% FBS with no treatment (negative control). The medium was renewed every 3 days, and on day 7, cells were stained with the differentiation marker, β‐III tubulin. Higher relative fluorescence intensities of β‐III tubulin (Figure 6) were found in cells treated with 2% (v/v) NHPPL (*p* < 0.05), 2% (v/v) HPPL (*p* < 0.01), and RA (*p* < 0.05), compared to the negative control. Thus, the HPPL nanofiltration did not affect its capacity to differentiate SH‐SY5Y cells into mature neurons.

#### 2.4.3 In vivo anti‐inflammatory activity in a TBI model

**FIGURE 7:**
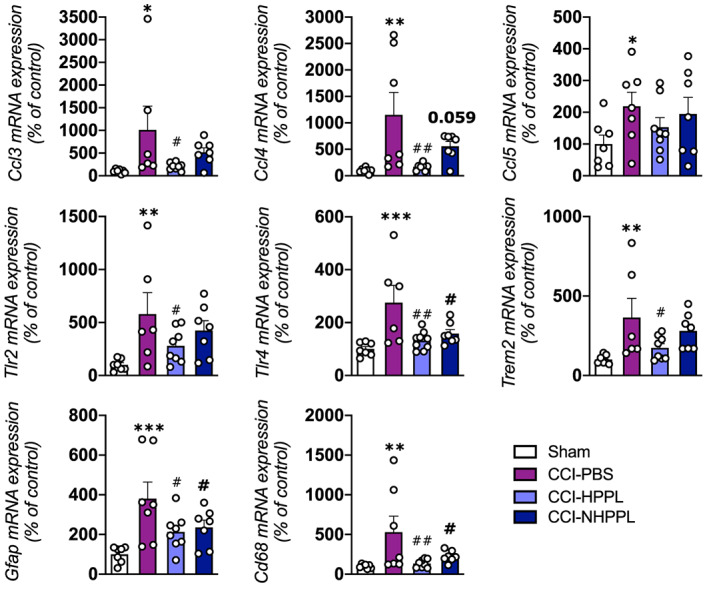
Modulation of neuroinflammatory markers in a mouse model of traumatic brain injury. A controlled cortical impact (CCI) was applied, and mice received either 60 μl heated platelet pellet lysate (HPPL), nanofiltered HPPL (NHPPL), or PBS, on three consecutive days by intranasal administration. Mice were sacrificed on day 7 post‐injury, the ipsilateral cortex was dissected out, and cytokine and glial marker mRNA levels were quantified by an RT‐qPCR. (*n* = 5–7 mice per group). Data are reported as the mean ± SEM.; *p < 0.05; **p < 0.01; ***p < 0.001 for CCI vs. Sham; ^#^ *p* < 0.05; ^##^ *p* < 0.01 for CCI‐PBS vs. CCI‐HPPL or CCI‐NHPPL by a one‐way ANOVA followed by Fisher's least significant difference test.

The controlled cortical impact (CCI) injury performed in the right hemisphere of mice was applied to induce gene expressions of several proinflammatory markers.^13^ Mice were treated with HPPL, NHPPL, and phosphate buffered‐saline (PBS) intranasally for three consecutive days on days 0–3. The administration of these materials was not associated with acute signs of toxicity. Mice were sacrificed on day 7 post‐injury. There was significant upregulation (*p* < 0.05) in the chemokine genes, *Ccl3*, *Ccl4*, and *Ccl5*, receptor genes, *Tlr2* and *Tlr4*, the astrocytic gene, *Gfap*, and the microglial marker genes, *Cd68* and *Trem2*, in injured mice compared to sham mice. Capacity of intranasal NHPPL to mitigate the neuroinflammation triggered by CCI was examined by comparing the differential gene expressions to PBS‐treated and non‐nanofiltered HPPL administration. Data (Figure 7) indicated significant downregulation of all tested genes (*p* < 0.05) in animals treated with HPPL compared to the PBS group, apart from *Ccl*5 (non‐significant decrease). NHPPL treatment induced a similar same trend as with HPPL, with significant downregulation (*p* < 0.05) of the *Tlr4*, *Cd68*, and *Gfap* proinflammatory genes, and relative decreases in *Ccl3*, *Ccl4*, *Ccl5*, *Tlr2*, and *Trem2*. There was no significant statistical difference in any of these markers between HPPL and NHPPL treatment. We concluded that NHPPL had intact functional activity to modulate proinflammatory markers post‐CCI injury.

## 3 DISCUSSION

There is a pressing need to develop safe neuroprotective and neuroregenerative biotherapies to counterbalance the overlapping cascade of physiopathological events associated with CNS diseases. These pathologies are characterized by neuroinflammation and oxidative stress with eventual synaptic alterations and loss of specific neuronal populations, which result in movement disorders, loss of cognitive functions, and/or behavioral impairments.^32^, ^33^, ^34^, ^35^ The complexity of most CNS diseases makes a single pharmacological approach unlikely to be therapeutically effective, supporting the emergence of cost‐effective multifaceted disease‐modifying strategies providing complementary therapeutic benefits to modulate neuroinflammatory processes, counterbalance oxidative stress, and control or reverse neuronal degeneration. Recent preclinical developments are demonstrating that brain administration of neurorestorative and neuroregenerative PC‐derived biomaterials has benefits in rodent models of PD,^9^, ^10^ AD,^11^, ^12^ stroke,^8^ and recently in TBI^13^ and ALS,^14^ opening concrete and pragmatic perspectives for an accessible and affordable disease‐modifying treatment of CNS diseases. The functional role of platelet‐derived HPL biomaterials is undoubtedly linked to their exceptional proteome comprising a physiological balance of functional trophic molecules that exert therapeutic benefits for cell growth and tissue regeneration.^17^, ^19^, ^24^, ^36^, ^37^, ^38^ As applied to treating brain disorders, the specific benefits of HPLs are most likely linked to the contents of neurotrophic growth factors, cytokines, neurotransmitters, and anti‐inflammatory and antioxidative biomolecules.^13^ We have obtained preclinical evidence that a tailor‐made purified heat‐treated HPL (termed HPPL) prepared from platelets isolated from clinical‐grade PCs is strongly neuroprotective in an MPTP mice model of PD.^10^ We found that it modulates immune responses, promotes wound healing, and improves cognitive function in mouse models of mild and moderate/severe TBI.^13^ These in vivo models confirmed the in vitro neuroprotective and neuroregenerative activities of HPPLs in primary neuronal cells and neuronal cell cultures.^15^, ^16^ To ensure safe brain administration, this HPPL is purposely depleted of the plasma protein compartment and is heat‐treated; such processing removes bulk proteins to avoid protein overload of the CSF, neurotoxic fibrinogen and prothrombotic factors and proteolytic enzymes.^10^ It is also vital to engineer HPLs that meet virus safety standards for clinical translation. As platelet lysates are made from human blood, virus safety is mandatory considering the history of massive viral transmissions by pooled blood products, and as standardized HPLs require the mixing of ca. 50–250 human PCs,^26^, ^39^, ^40^ inevitably increasing the risk of virus contamination.^25^, ^26^ Virus safety is especially vital as these platelet materials will be administered to new patient populations who, by contrast to hemophiliacs and immune‐deficient individuals who need life‐long transfusions, are not typically exposed to human blood products.^25^, ^26^ Human blood and PCs can be contaminated by HIV and HBV, and several flaviviruses (e.g., HCV, ZIKV, DENV, or WNV) and coronaviruses exerting neuropathological effects.^41^, ^42^ Despite stringent donor selection and individual donation virus testing, PCs may be contaminated by window period donations and untested viruses,^43^, ^44^ which may be present in HPL preparations if no additional virus‐safety measures are taken.

Only a few studies have examined the impacts of virus/pathogen‐reduction treatments on the neuroprotective activities of HPLs. One of our previous studies showed that HPPL made from PCs subjected to psoralen/ultraviolet A (UVA) photochemical pathogen‐reduction treatment^43^, ^44^ maintained its neuroprotective activity in various neuronal cell models.^16^ However, that treatment cannot inactivate all possible blood‐borne viruses, especially some non‐enveloped viruses.^43^ Our study here identified for the first time that HPPL could be subjected to a dedicated nanofiltration process using 19‐nm pore‐size cartridges. Low‐protein content‐HPPL was prepared from PCs centrifuged to pelletize the platelets and remove the plasma. HPPL was pre‐filtered using 0.2‐ and 0.1‐μm filters to decrease any risk of nanofilter clogging, a common issue with complex protein solutions. In this study, over 20 ml of HPPL could be filtered through the 0.001‐m^2^ Planova‐20N system in approximately 3.5 h at a constant flow‐rate of 0.1 ml/min and low pressure, corresponding to over 20 L of industrial‐scale HPPL using a commercial 1‐m^2^ Planova‐20N cartridge (and over 80 L using a commercial 4‐m^2^ Planova‐20N). We expect, based on preclinical data^10^, ^13^, ^14^ and considering the volume of the cerebrovascular fluid in humans, that a 20 L batch would allow to treat approximately 80 patients per year using a treatment dose of 5 ml per patient per week.

The virus‐removal capacity, assessed by immuno‐qPCR detection of MVP was greater than 5 log units, thus demonstrating the efficiency of nanofiltration for eliminating even small viruses. Nanofiltration can remove a wide range of viruses and has been instrumental in improving the safety of industrial plasma‐derived products thanks to a removal mechanism that is determined by the size of the virus relative to that of the membrane.^31^, ^45^ In the biological product industry, including blood plasma‐derived products, nanofiltration has indeed proven to be a robust method of virus removal.^30^ Typically, the filters used for licensed products have a mean pore size of 35, 19, or 15 nm^30^, ^31^, ^45^; the selection of the nanofilter cut‐off depends upon the characteristics (especially the size) of the proteins to be subjected to nanofiltration to avoid filter clogging (blood protein size ranges roughly from 3 to 15 nm). A comprehensive review indicates that the Planova 20N nanofilter with a mean pore size of 19 ± 2 nm is capable to remove all known blood‐borne viruses, including the small non‐enveloped parvovirus B19 (B19V) with a size of 18–26 nm and hepatitis A virus with a size of 26–29 nm from various industrial fractionated human plasma products.^30^ The capacity of Planova 20N to robustly remove B19V has also been demonstrated by microscopic observations of the nanofilter.^47^ Finally, another small blood‐borne non‐enveloped virus, hepatitis E virus (HEV), with a size of 27–34 nm, was also demonstrated to be removed by Planova 20N.^48^ Therefore, all known blood‐borne viruses of clinical relevance are removed by Planova 20N, providing a large safety margin to NHPPL. As such, Planova‐20N should robustly remove all known blood‐borne viruses, either enveloped or non‐enveloped.

Compared to the non‐nanofiltered product, the NHPPL was depleted of EVs, including pro‐coagulant PS‐expressing EVs, while the protein composition was largely preserved, as revealed by LC/MS‐MS proteomics. Most importantly, the nanofiltration step did not alter the neuroprotective, neurorestorative, or anti‐inflammatory properties of HPPL in in vitro cellular models of PD and TBI, and upon i.n. administration, in a mouse model of TBI. These data are vital for future clinical assessments, regulatory approval, and licensing of virally safe and functional HPL preparations produced from pools of human PCs. Protein quantification data and ELISA assessments identified decreases in total proteins and growth factors, such as PDGD‐AB and VEGF, which could reflect dilution occurring during nanofiltration and/or non‐specific adsorption onto the Planova‐20N hollow‐fiber membranes. These growth factors were selected because they are important neurotrophins which we assessed in our previous studies to characterize HPPL.^10^, ^13^, ^16^, ^22^, ^49^ Besides, their molecular mass (VEGF: ca. 40 kDa; PDGF: ca. 30 kDa; and EGF: ca. 6 kDa) is representative of that of the HPPL trophic factors a parameter that is relevant to assess a virus reduction technology based on size‐exclusion. Also, these growth factors could potentially be associated with EVs and other lipid vesicles present in HPPL and removed by the filtration sequence. In addition, we have restricted the ELISA determination to these three growth factors considering that a more extensive characterization of the impacts of the nanofiltration process has been performed by proteomics.

The proteomic analysis identified that the heat‐treatment previously found to improve or normalize the safety and efficacy of HPPL^10^, ^16^ led to even more pronounced removal of fibrinogen and thrombogenic factors. A decrease in total protein number was detectable after nanofiltration, but over 80% of them were common. Proteins contributing to essential functions of HPPL for brain therapy, such as antioxidants, cytokines, and neurotrophic factors, were well preserved in the NHPPL, with high similarity in the BPs involved. The KEGG database showed that the filtration/nanofiltration sequence removed additional proteins associated with disease‐associated pathways. Those mainly included bacterial/viral/parasitic infection‐related pathways and proteins associated with cancerous pathologies. This decreased abundance may reflect the removal of PEVs, as discussed below. Indeed, PEVs are associated with biological reactions during infections or during cancer progression.^50^ Also, platelets in the blood circulation are known to contribute to defense mechanisms against various pathogens and can undergo activation that triggers multiple signaling pathways through its secretome products and the recruitment of immune cells.^46^, ^51^ Furthermore, the KEGG pathway analysis revealed decreasing abundances of some proteins involved in coagulation through the filtrations. This observation supports that the NHPPL should have an even lower risk of thrombogenicity than HPPL when administered to the brain.

Another informative aspect was the impact of nanofiltration on the removal of EVs. Most proteins removed were associated with extracellular exosomes. EVs can be classified as “small,” “medium,” or “large” according to their size.^52^ In platelet lysates, EVs have a size ranging between approximately 50 and 300 nm.^21^ Planova 20N thus removed particles larger than its 19‐nm mean pore size. Proteins associated with EVs still detected after nanofiltration may suggest their presence in a soluble form or a possible disruption of the EVs into smaller entities during the nanofiltration process. EVs removal was supported by DLS, NTA, and functional prothrombotic and procoagulant EV assays. DLS analysis showed decreased main size distribution of particles from approximately 280 nm in the HPPL to approximately 12 nm in the NHPPL. The filtration sequence decreased the EV numbers by nearly 99% (2 logs), as indicated by NTA. The value may be underestimated due to the presence of proteins in the ca. 5–12‐nm range not being removed by nanofiltration and detected by DLS and NTA. In addition, the MP‐activity functional assay, a capture assay that quantifies functional PS‐expressing EVs, was used to evaluate the impact of nanofiltration on these specific EVs. A previous study showed that treatment of HPPL at 56°C for 30 min lowers the content in functional PS‐expressing EVs.^53^ We found here that the amount of EVs bearing PS in NHPPL was significantly decreased compared to HPPL. The STA‐procoagulant‐PPL assay, which is a phospholipid procoagulant‐dependent clotting time assay, suggested removal of procoagulant PEVs, as indicated by a robust prolongation in the coagulation time from the NHPPL compared to the HPPL. The decrease in PS‐expressing EVs afforded by nanofiltration should limit even more the risks of coagulation and therefore further improve the safety of NHPPL for brain administration.

An in vitro study was performed using LUHMES cells as a PD model to unveil any impact of nanofiltration on the neuroprotective activity of HPPL. NHPPL at a dose of 5% (v/v) was not toxic and maintained a capacity to protect cells from the erastin neurotoxic drug. Cells remained viable 24 h post‐treatment, similar to that achieved with the HPPL in our previous studies.^10^, ^16^ We also evaluated the ability of NHPPL to support cell maturation, as was observed previously with HPPL.^16^, ^22^, Our study using the SH‐SY5Y neuroblastoma cell line revealed that after 1 week of treatment with 2% NHPPL, cells strongly expressed the β‐III tubulin differentiation marker, similar to what was observed with the HPPL and RA‐positive controls. Thus, NHPPL maintained a capacity to promote cell differentiation and maturation, which is essential to counterbalance progressive neuronal degeneration.

We performed CCI, an in vivo model of mild TBI,^54^ to assess the capacity of the NHPPL to modulate inflammatory markers after a concussion, as found previously with the non‐nanofiltered HPPL.^13^ HPPL and NHPPL were delivered through an i.n. route over three consecutive days and their anti‐inflammatory actions in ipsilateral cortical tissues were assessed 7 days after injury. Both were administered at a dose of 60 μl/day for a total dose of 180 μl over 3 days. This daily dosing was selected based on our previous in vivo studies using HPPL in PD^10^ and TBI^13^ mouse models. Also, the in vitro functional activity of the HPPL and NHHPL batches used here was consistent with that observed with HPPL in our previous studies.^10^, ^16^, ^22^ Gene expression data showed that the overexpression of inflammatory markers in non‐treated TBI mice was significantly downregulated in mice treated with both the HPPL and NHPPL. This result suggests that, after nanofiltration, the HPPL retained the anti‐inflammatory potential in TBI models. In addition, compared to our previous study, where both topical and intranasal administrations were performed,^13^ our current results demonstrate that in this TBI mouse model, i.n. administration of the NHPPL alone was effective. This is clinically vital for the treatment of TBI where there is no brain access and intranasal administration would be the only feasible delivery option. Our data indicate that removing PEVs from HPPL by nanofiltration does not affect the neuroprotective activity, suggesting that these EVs are not essential to the neuroprotective and anti‐inflammatory activity of this biomaterial. However, EVs from other HPLs promote cell growth and migration of neuronal cells and stimulate network formation in primary neuronal cultures.^21^ Our in vivo evaluation has two limitations. First it was conducted, for comparative purposes with our previous work,^13^ in male mice only, while females may experience distinct pathophysiological impacts from TBI that are understudied.^55^ This implies that further TBI work should include evaluation in female mice. Second, our study did not include a histological assessment of injured tissues and behavioral tests, which would be valuable to confirm the anti‐inflammatory effects of NHPPL using various markers such as GFAP and Iba‐1. In addition, this study does not exclude a possible impact, either negative or positive, of the nanofiltration of HPPL on other important pathophysiological factors for TBI treatment, such as protection of synaptic markers, antioxidative activity, or behavioral recovery.^13^

## 4 MATERIALS AND METHODS

### 4.1 Source of PCs and HPPL preparation

The Institutional Review Board of Taipei Medical University, Taipei, Taiwan approved the study (TMU‐JIRB no. 201802052). Collection and processing of the PC into HPPL are described in Supporting Information S1.

### 4.2 Nanofiltration processes of HPPL

HPPL (ca. 25 ml) was pre‐filtered through 0.2‐μm PN 4612 and 0.1‐μm PN 4611 filters (Pall Life Science), then 20 ml was directly filtered through 0.001‐m^2^ Planova‐20N (19 ± 2 nm pore size; Asahi Kasei). Nanofiltration was performed at 22 ± 0.5°C using an AKTA system (GE Healthcare Life Sciences) at constant flow rate of 0.1 ml/min, monitoring the protein adsorption at 280 nm of the Planova‐20N filtrate and the transmembrane pressure. The filtrate was recovered, aliquoted, and stored at −80°C for several weeks or months until all analyses including the in vitro and in vivo functional assays.

### 4.3 Virus‐removal assessment during nanofiltration

An immuno‐qPCR‐based assay using an MVM‐MVP kit with a MOCK‐V solution (Cygnus Technologies) was used to evaluate the virus‐removal efficiency of nanofiltration (Supporting Information S1).

### 4.4 Evaluation of total protein, trophic factors, proteomic, and bioinformatics analyses

We determined the total protein concentration and growth factor content and performed proteomics analysis by LC‐MS/MS as described before^13^, ^56^ and in Supporting Information S1.

### 4.5 Determination of physical and functional properties of the EVs

DLS (Zetasizer, Malvern) and NTA (NanoSight NS300, Malvern) were used to respectively determine the main size distribution and concentration of EVs. For NTA analysis, HPPL was diluted 2 × 10^3^‐fold, and both 0.2–0.1 μm‐filtered and NHPPL were diluted 100‐fold using 0.1 μm filtered PBS. An STA‐procoagulant‐phospholipid (Diagnostica, Stago) assay was performed to determine the global procoagulant activity associated with EVs in the HPPL before and after nanofiltration.^57^ The pro‐thrombogenic activity associated with the presence of EVs expressing functional PS was determined by the functional Zymuphen microparticle (MP)‐activity assay (Hyphen BioMed) as described previously.^57^

### 4.6 Assessment of in vitro functionality of neuronal cells

#### 4.6.1 In vitro neuroprotective activity of LUHMES cells against erastin

Experimental LUHMES cells cultures are provided in Supporting Information S1. On the fifth day of differentiation, LUHMES cells were pretreated with 5% (v/v) of the crude HPPL, 0.2–0.1‐μm pre‐filtered HPPL, or NHPPL. After 1 h, a dose of 1 μM erastin for neurotoxic stimulation was added. Cell viability was assessed after 24 h of incubation using the CCK‐8 assay (Sigma‐Aldrich). The absorbance was measured at 450 nm. Cell viability was expressed as a percentage of viable cells compared to untreated cells considered as the 100% viability control.

#### 4.6.2 Differentiation of human SH‐SY5Y neuroblastoma cells

SH‐SY5Y neuroblastoma cells were maintained in high‐glucose DMEM (Hyclone Laboratories) supplemented with 100 U/ml penicillin, 100 U/ml streptomycin (Gibco, Life Technology), and 10% FBS (Gibco). Cells were incubated at 37°C in a controlled‐humidity 5% CO_2_ incubator. To stimulate cell maturation, SH‐SY5Y cells were seeded in six‐well plates at a density of 4 × 10^4^ cells/well. The medium was changed after 24 h, and then 1 μM RA (positive control), 2% (v/v) of crude HPPL, and 2% (v/v) NHPPL were added to dedicated wells. The medium was changed every 3 days, and on day 7, cells were immunostained. The capacity to induce SH‐SY5Y cell differentiation was examined by β‐III tubulin (Abcam: cat no. Ab18207; 54 kDa 1:500 dilution) fluorescence staining.

#### 4.6.3 Assessment of anti‐inflammatory functionality in a TBI mouse model *CCI model*

The animal study was conducted according to ethical guidelines and with a protocol approved by the animal facility center of Taipei Medical University (TMU) (application no. LAC 2020‐0042). Adult male C57/BL6 mice (aged 8–12 weeks, weighing 20–30 g) were purchased from the Taiwan National Laboratory Animal Center. Mice were housed at the TMU animal facility under a controlled dark (12 h)‐light (12 h) cycle. A mild TBI was induced in mice, using a eCCI‐6.3 (Custom Design & Fabrication) exactly as before.^13^ The treatment using HPPL, NHPPL, and PBS (vehicle control) was administrated approximately 2 h post‐injury by intranasal (i.n.) administration. Test materials (60 μl) were delivered using a pipette by alternating the nostrils and maintaining 5‐min intervals between each 20‐μl administration. This treatment was repeated on three consecutive days, with each mouse receiving 180 μl in total. On day 7, mice were sacrificed by cervical dislocation, the brains were quickly taken and rinsed in cold PBS, and the injured area of the ipsilateral cortex was collected using a 4.0‐mm biopsy punch. Samples were then frozen in liquid nitrogen until further gene expression analysis by qPCR (Supporting Information S1).

### 4.7 Statistical analysis

Statistical analyses were performed using GraphPad Prism software vers. 6.0, and data are expressed as a mean ± standard deviation (SD) or standard error of the mean (SEM). A one‐way analysis of variance (ANOVA) followed by Fisher's least significant difference (LSD) test was performed for comparison, and differences were considered significant at *p* < 0.05.

## 5 CONCLUSION

This study provides important new information for bioengineering and clinical translation of an HPL biotherapy of neurodegenerative disorders and brain trauma. First, we demonstrate that a scalable nanofiltration step using 19‐nm filters dedicated for virus removal can be incorporated in the process of production of the platelet lysate without technical difficulties nor risks of nanofilter clogging, while also ensuring robust (>5 log) removal of a small virus model. This is vital for clinical application as a guarantee of sufficient margin of virus safety will be needed for clinical application. Second, the nanofiltration process did not affect the in vitro neuroprotective and repair capacity nor the in vivo anti‐inflammatory activity of the HPPL. This was also crucial to verify as a loss of functional activity would have definitely disqualified the use of this virus reduction technology for this application. Third, while we were not surprised by the removal of the EVs by the nanofiltration step,^49^, ^57^ we were expecting a decrease in the functional activity of HPPL associated with EV removal, which was actually not observed. This showed that the EV‐depleted HPPL protein compartment has inherent neuroprotective, repair and anti‐inflammatory properties by itself, which is an important new information. Fourth, the in vivo study showed that administration of HPPL and NHPPL by intranasal delivery alone was effective to exert an anti‐inflammatory activity in the TBI model. In our previous studies in TBI models with created brain access,^13^ the HPPL was first applied topically 1 h after injury onto the wound, followed by 6 days of intranasal delivery. The current study reveals that i.n. alone could be an option for the anti‐inflammatory treatment of TBI without brain access, an information that is vital for clinical translation.

The limitations of our study include the fact that the anti‐inflammatory effects of HPPL and NHPPL has not been evaluated in a TBI model using female mice, nor the protection of dopaminergic neurons in a PD mice model. Besides, we did not establish yet the optimum delivery schedule of the NHPPL to the brain, considering also that it will likely depend upon the mode of delivery (intracranial, intranasal, topical and/or intracerebroventricular) that can affect the effective dose delivered to the pathological site. Finally, although we show an improvement in safety parameters, such as due to the removal of pro‐coagulant factors, associated with nanofiltration, only long‐term administration in pre‐clinical models will allow assessing the safety of NHPPL. However, jointly, the cumulative data unveiled in our study, are vital to support future translational developments of this biotherapy of brain disorders.

## AUTHOR CONTRIBUTIONS

**Liling Delila:** Data curation (lead); formal analysis (equal); investigation (lead); methodology (equal); writing – original draft (lead); writing – review and editing (lead). **Ouada Nebie:** Data curation (supporting); formal analysis (equal); investigation (supporting); methodology (equal); writing – original draft (supporting); writing – review and editing (supporting). **Nhi Thao Ngoc Le:** Data curation (supporting); formal analysis (supporting); investigation (supporting); methodology (supporting). **Lassina Barro:** Data curation (supporting); formal analysis (supporting); investigation (supporting); methodology (supporting); writing – original draft (supporting). **Ming‐Li Chou:** Data curation (supporting); investigation (supporting); writing – review and editing (supporting). **Yu‐Wen Wu:** Methodology (supporting); writing – review and editing (supporting). **Naoto Watanabe:** Data curation (supporting); methodology (supporting); writing – review and editing (supporting). **Masayasu Takahara:** Methodology (supporting); writing – review and editing (supporting). **Luc Buée:** Writing – review and editing (equal). **David Blum:** Investigation (supporting); methodology (equal); supervision (supporting); writing – original draft (supporting); writing – review and editing (supporting). **David Devos:** Investigation (supporting); supervision (equal); writing – review and editing (supporting). **Thierry Burnouf:** Conceptualization (lead); formal analysis (equal); funding acquisition (lead); investigation (supporting); methodology (equal); project administration (lead); resources (lead); supervision (lead); writing – original draft (lead); writing – review and editing (lead).

## CONFLICTS OF INTEREST

Naoto Watanabe and Masayasu Takahara are employees of Asahi Kasei Medical. Thierry Burnouf and David Devos are named as inventors of patent applications owned by their respective universities and institutions and are founders of Invenis Biotherapies. The other authors have no commercial, proprietary, or financial interest in the products or companies described in this article.

6

### PEER REVIEW

The peer review history for this article is available at https://publons.com/publon/10.1002/btm2.10360.

## Supporting information


**Appendix S1** Supporting Information

## Data Availability

The datasets generated during and/or analysed during the current study are available from the corresponding author upon reasonable request.
