# Supplementary material for: Neuroprotective activity of a virus‐safe nanofiltered human platelet lysate depleted of extracellular vesicles in Parkinson's disease and traumatic brain injury models
Source: Bioeng Transl Med. 2022 Jul 12;8(1):e10360. doi: 10.1002/btm2.10360 (PMC9842020; doi:10.1002/btm2.10360)
Supplement: Supplementary file 1 — Appendix S1 Supporting Information [file BTM2-8-e10360-s001.docx]

**Appendix 1 : Supplementary file**

1. **Supplementary Figures**

**
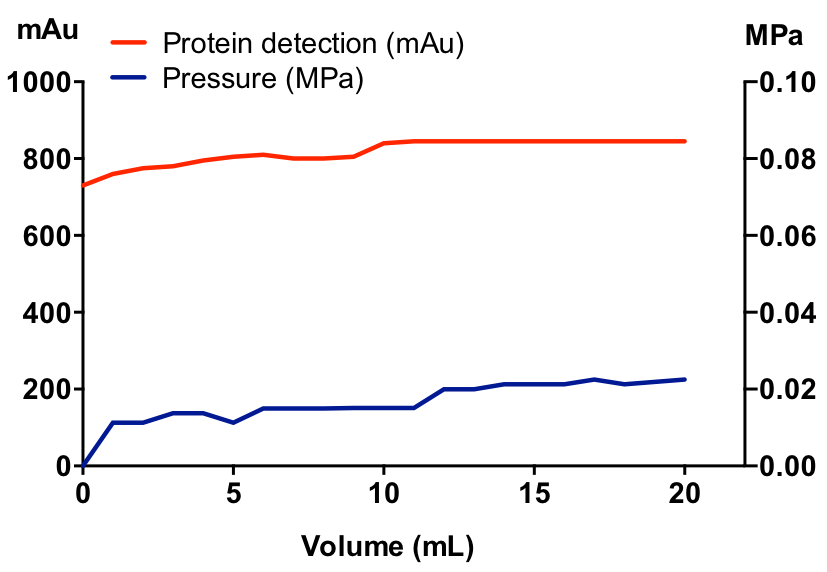
**

**Supplementary Figure 1.** Filtration curve of human platelet pellet lysate (HPPL) recorded by AKTA system, around 20 mL were collected through Planova-20N 0.0001-m^2^ without reaching the maximum pressure

**
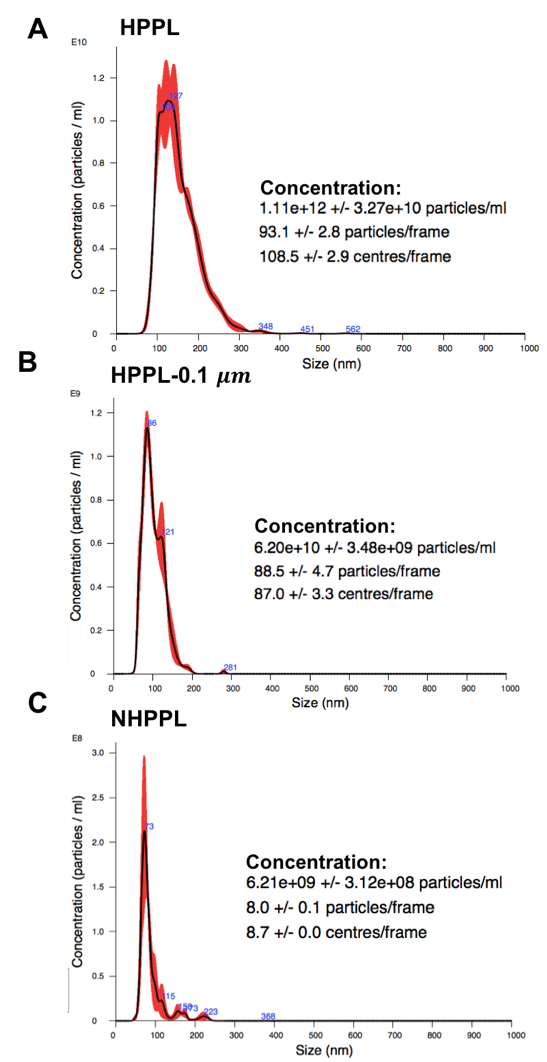
**

**Supplementary Figure 2.** Platelet extracellular vesicle (PEV) numbers in heat-treated platelet pellet lysate (HPPL), after 0.2~0.1-µm filtration (HPPL-0.1 µm), and in nanofiltered HPPL (NHPPL) determined by a nanoparticle tracking analysis (NTA). HPPLs were diluted 2×10^3^-fold, 0.2~0.1-µm filtration and NHPPL, using 0.1-µm-filtered PBS prior to the analysis.

**
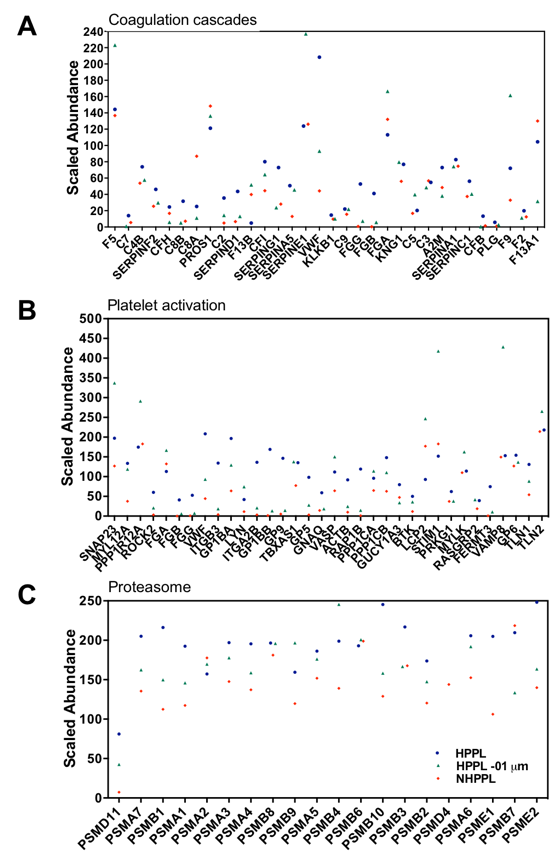
**

**Supplementary Figure 3**. Relative abundances of proteins associated with KEGG pathways of complement and coagulation cascades (A), platelet activation (B), and proteasome (C) after 0.2~0.1-μm filtration and nanofiltration (NHPPL). **Abbreviations:** coagulation factor V (F5); complement component (C7); complement C4-B (C4B); alpha-2-antiplasmin (SERPINF2); complement factor (CFH); complement component beta (C8B); complement component alpha (C8A); vitamin K-dependent protein S (PROS1); complement C2 (C2); heparin cofactor 2 (SERPIND1); coagulation factor XIII B (F13B); complement factor-I (CFI); plasma protease C1 (SERPING1); plasma serine protease inhibitor (SERPINA5); plasminogen activator inhibitor 1 (SERPINE1); von Willebrand factor (VWF); plasma kallikrein (KLKB1); complement component C9 (C9); fibrinogen gamma (FGG); fibrinogen beta (FGB); fibrinogen alpha chain (FGA); kininogen-1 (KNG1); complement C5 (C5); complement C3 (C3); alpha-2-macroglobulin (A2M); alpha-1-antitrypsin (SERPINA1); antithrombin-III (SERPINC1); complement factor B (CFB); plasminogen (PLG); coagulation factor IX (F9); prothrombin (F2); coagulation factor XIII-A (F13A1).

1. **Supplementary Tables**

Supplementary Table 1: List of the primers used in this study

| **Name** | **Forward primer** | **Reverse primer** |
| --- | --- | --- |
| *Cyclophilin* | agcatacaggtcctggcatc | ttcaccttcccaaagaccac |
| *Ccl3* | tgcccttgctgttcttctct | gtggaatcttccggctgtag |
| *Ccl4* | gccctctctctcctcttgct | gagggtcagagcccattg |
| *Ccl5* | ctcactgcagccgccctctg | ccgagccatatggtgaggcagg |
| *Tlr2* | ggggcttcacttctctgctt | agcatcctctgcgatttgacg |
| *Tlr4* | ggactctgatcatggcactg | ctgatccatgcattggtaggt |
| *Trem2* | cgagaggctgaggtcctg | tctccagcatcttggtcatcta |
| *Gfap* | cgcgaacaggaagagcgcca | gtggcgggccatctcctcct |
| *Cd68* | gacctacatcagagcccgagt | cgccatgaatgtccactg |

**Supplementary Table 2: List of neurotrophic factors, cytokines, antioxidants found in the common proteins list**

| **Accession** | **Protein FDR Confidence: Combined** | **Description** |
| --- | --- | --- |
| P02776 | High | Platelet factor 4 OS=Homo sapiens OX=9606 GN=PF4 PE=1 SV=2 |
| P00450 | High | Ceruloplasmin OS=Homo sapiens OX=9606 GN=CP PE=1 SV=1 |
| P00441 | High | Superoxide dismutase [Cu-Zn] OS=Homo sapiens OX=9606 GN=SOD1 PE=1 SV=2 |
| P55145 | High | Mesencephalic astrocyte-derived neurotrophic factor OS=Homo sapiens OX=9606 GN=MANF PE=1 SV=3 |
| P04040 | High | Catalase OS=Homo sapiens OX=9606 GN=CAT PE=1 SV=3 |
| P00390 | High | Glutathione reductase, mitochondrial OS=Homo sapiens OX=9606 GN=GSR PE=1 SV=2 |
| Q14766 | High | Latent-transforming growth factor beta-binding protein 1 OS=Homo sapiens OX=9606 GN=LTBP1 PE=1 SV=4 |
| P01137 | High | Transforming growth factor beta-1 proprotein OS=Homo sapiens OX=9606 GN=TGFB1 PE=1 SV=2 |
| P04179 | High | Superoxide dismutase [Mn], mitochondrial OS=Homo sapiens OX=9606 GN=SOD2 PE=1 SV=3 |
| P10720 | High | Platelet factor 4 variant OS=Homo sapiens OX=9606 GN=PF4V1 PE=1 SV=1 |
| P35858 | High | Insulin-like growth factor-binding protein complex acid labile subunit OS=Homo sapiens OX=9606 GN=IGFALS PE=1 SV=1 |
| Q969H8 | High | Myeloid-derived growth factor OS=Homo sapiens OX=9606 GN=MYDGF PE=1 SV=1 |
| Q9UBC2 | High | Epidermal growth factor receptor substrate 15-like 1 OS=Homo sapiens OX=9606 GN=EPS15L1 PE=1 SV=1 |
| P48637 | High | Glutathione synthetase OS=Homo sapiens OX=9606 GN=GSS PE=1 SV=1 |
| Q9UNF0 | High | Protein kinase C and casein kinase substrate in neurons protein 2 OS=Homo sapiens OX=9606 GN=PACSIN2 PE=1 SV=2 |
| P22352 | High | Glutathione peroxidase 3 OS=Homo sapiens OX=9606 GN=GPX3 PE=1 SV=2 |
| P78417 | High | Glutathione S-transferase omega-1 OS=Homo sapiens OX=9606 GN=GSTO1 PE=1 SV=2 |
| O60234 | High | Glia maturation factor gamma OS=Homo sapiens OX=9606 GN=GMFG PE=1 SV=1 |
| P60983 | High | Glia maturation factor beta OS=Homo sapiens OX=9606 GN=GMFB PE=1 SV=2 |
| P10644 | High | cAMP-dependent protein kinase type I-alpha regulatory subunit OS=Homo sapiens OX=9606 GN=PRKAR1A PE=1 SV=1 |
| P42566 | High | Epidermal growth factor receptor substrate 15 OS=Homo sapiens OX=9606 GN=EPS15 PE=1 SV=2 |
| P51858 | High | Hepatoma-derived growth factor OS=Homo sapiens OX=9606 GN=HDGF PE=1 SV=1 |
| O14964 | High | Hepatocyte growth factor-regulated tyrosine kinase substrate OS=Homo sapiens OX=9606 GN=HGS PE=1 SV=1 |
| O95825 | High | Quinone oxidoreductase-like protein 1 OS=Homo sapiens OX=9606 GN=CRYZL1 PE=1 SV=2 |
| Q16775 | High | Hydroxyacylglutathione hydrolase, mitochondrial OS=Homo sapiens OX=9606 GN=HAGH PE=1 SV=2 |
| P09211 | High | Glutathione S-transferase P OS=Homo sapiens OX=9606 GN=GSTP1 PE=1 SV=2 |
| P07203 | High | Glutathione peroxidase 1 OS=Homo sapiens OX=9606 GN=GPX1 PE=1 SV=4 |
| P49767 | High | Vascular endothelial growth factor C OS=Homo sapiens OX=9606 GN=VEGFC PE=1 SV=1 |
| P62993 | High | Growth factor receptor-bound protein 2 OS=Homo sapiens OX=9606 GN=GRB2 PE=1 SV=1 |
| Q04756 | High | Hepatocyte growth factor activator OS=Homo sapiens OX=9606 GN=HGFAC PE=1 SV=1 |
| P68402 | High | Platelet-activating factor acetylhydrolase IB subunit beta OS=Homo sapiens OX=9606 GN=PAFAH1B2 PE=1 SV=1 |

**3. Supplementary materials and methods**

**3.1 PC collection**

Clinical-grade PCs were obtained by centrifugation of anticoagulated non-leucoreduced whole blood, collected from donors at the Taipei Blood Center (Guandu, Taiwan). The PCs were transported (90 min) to the laboratory under controlled temperature and processed within 24 h of receipt, corresponding to 6~7 days from the day of collection. The blood cell count in each PC was determined using an ABC Vet blood cell count (ABC Diagnostics, Montpellier, France). For this study, several PC and HPPL batches were pooled into one representative batch made from 50 donors, and these were verified to meet the protein and EV product quality specifications of batches used in our previous studies [1-3].

3.2 **HPPL preparation**

HPPL was prepared following our standard laboratory protocols as described previously [1, 3]. Pooled PCs were centrifuged to pelletize the platelets at 3000 ×*g* for 30 min at 22±$2$ °C. The pellet was resuspended using PBS, subjected to three freeze (-80±1 °C)-thaw (37±1 °C) cycles to break the platelets, and centrifuged at 4500 ×*g* for 30 min at 22±2 °C to remove insoluble materials, after which the supernatant was collected. The supernatant was further purified by 56±1 °C heat treatment, cooled at 4 °C, and centrifuged at 10^4^ ×*g* for 15 min at 4±2 °C to obtain the HPPL. The HPPL was aliquoted and stored at -80 °C until use. Prior to each evaluation, samples were thawed at 37±$1$°C and centrifuged at 10^4^ ×*g* for 15 min at 4±$1$ °C to remove any insoluble proteins.

**3.3 Virus-removal assessment during nanofiltration**

**3.3.1 Spiking experiment of MVM-MVP**

MVP, composed of small, stable, non-enveloped parvovirus-like particles (20~22 nm), was spiked into 4 mL of HPPL, pre-filtered with 0.2- and 0.1-µm filters, to a final total concentration of 10^10^ MVPs/mL. A small aliquot of the spiked HPPL was taken as a positive control and either immediately frozen at -80 °C after spiking or kept on the bench for the duration of nanofiltration. The MVP-spiked HPPL was then passed through a nanofilter consisting of a single hollow-fiber Planova-20N 0.0001-m^2^ membrane that was specially manufactured for these experiments by Asahi-Kasei. Nanofiltration was conducted at a constant pressure of 0.08 MPa using compressed air. The non-nanofiltered HPPL, the 0.2~0.1-µm pre-filtered spiked HPPL control, and the NHPPL were stored at -80 °C prior to further analyses. The spiking experiment was done in duplicate to assess the log reduction clearance of MVPs by an immuno-qPCR analysis.

**3.3.2 Immuno-qPCR and MVP clearance factor**

The immuno-qPCR test was performed following the manufacturer’s (Cygnus Technologies) recommendations as described previously [4]. Briefly, prior to the immuno-qPCR, reagents were allowed to warm up to room temperature (22 °C) for 30 min. Serial dilutions of the MVP stock (10^12^ MVPs/mL) were prepared and used as standards. A monoclonal antibody (mAb)-coated microplate was first washed with 400 μL of washing buffer and allowed to soak for 10 min. After drying, 100 μL of samples and the standard were added in duplicate and incubated for 30 min at 37±1 °C. Then, the solutions were removed, and the wells were washed. An MVP detector antibody (100 μL) was added to each well and incubated for 30 min at 37 °C. At the end of incubation, the wells were washed with buffers 1 and 2 provided by the supplier and dried. The recovery solution (50 μL) was added and incubated at room temperature for 5 min. Recovered samples were then mixed well, and 5 μL was transferred to a qPCR plate. TaqMan master mix was prepared as recommended by the provider, and oligomers, the 6-FAM probe, and DNase/RNase-free water were used. Then, 20 μL of this preparation was distributed to the qPCR-well plate, sealed, and centrifuged at 1000 ×*g* for 2 min. Samples were run on a StepOne™ Real-Time PCR system as recommended by Cygnus Technologies). At the end of amplification, the cycle threshold (Ct) of the standard was used to generate a curve, and the Ct of the samples was then plotted against the logarithmic standard curve. The LRV was quantified using the following formula: LRV = [Cl*Vl/(Cf*Vf)], where Cl is the loaded concentration, Vl is the volume loaded, Cf is the final concentration after nanofiltration, and Vf is the final volume.

**3.4 Evaluation of total protein and trophic factors**

We determined the total protein concentration of the HPPL by a Pierce^TM^ bicinchoninic acid (BCA) assay (ThermoFisher Scientific, Waltham, MA, USA), using a range of 25~2000 μg/mL of BSA as a standard. Contents of PDGF-AB, EGF, and VEGF were quantified by an enzyme-linked immunosorbent assay (ELISA; DuoSet R&D Systems, Minneapolis, MN, USA) following the manufacturer’s protocol and as previously described [5, 6].

**3.5 Proteomic analysis**

**3.5.1 Samples preparation**

Samples were treated with acetone pre-cooled to -20 °C using a sample/acetone ratio (v/v) of 1/4. The mixture was left overnight at -20 °C, then centrifuged at 15,000 ×*g* for 10 min at 4 °C. The pellet was washed twice with cold acetone in water (1/4) and centrifuged at 13,000 ×*g* for 10 min at 4 °C. The supernatant was discarded, while the pellet was air-dried and re-suspended in 6 M urea. The protein content was quantified by a BCA protein assay kit, and 20 μg of protein was used for the proteomics analysis

**3.5.2 Data analysis**

For each raw data file recorded by mass spectrometry (MS), peak lists were generated using Data Analysis vers. 4.3 (LC-quadrupole time of flight (QTOF); Bruker Daltonics, Billerica, MA, USA) and Proteome Discoverer vers. 2.2 or 2.4 (LTQ Orbitrap; ThermoFisher Scientific). The human UniProt Swiss-Prot database (with 20,431 annotated proteins, release 2019.07) was used. The false discovery rate (FDR) of the spectrum and protein matching was set to 1%. We used the DAVID (The Database for Annotation, Visualization and Integrated Discovery) functional annotation tool (https://david.ncifcrf.gov/) to annotate and enrich corresponding genes by GO and KEGG pathways. A Venn diagram was drawn using the Python matplotlib-venn package.

**3.6 EVs functional activity characterization**

**3.6.1 STA-procoagulant-phospholipid**

An STA-procoagulant-phospholipid (Diagnostica, Stago, Asnières, France) assay was performed to determine the global procoagulant activity associated with EVs in the HPPL before and after nanofiltration [7]. Briefly, 25 µL of citrated human plasma depleted of phospholipids was added to 25 µL HPPL and NHPPL and then incubated at 22±2 °C for 120 s. To initiate coagulation, 100 µL of activated factor X (FXa) was added, and an STA compact automatic coagulometer was used to measure the clotting time. Positive and negative controls provided in the kit were assessed as recommended.

**3.6.2 Microparticle (MP)-activity assay**

The pro-thrombogenic activity associated with the presence of EVs expressing functional PS was determined by the functional Zymuphen MP-activity assay (Hyphen BioMed, Paris, France) as described previously [8]. Briefly, 100 µL of HPPL, 0.2~0.1-µm-filtered HPPL, and NHPPL were respectively pre-diluted 3×10^4^-, 2.5×10^3^-, and 10^2^-fold in the sample diluent, and then added to an Annexin-precoated microplate and incubated for 60 min at 37 °C. To activate prothrombin into thrombin, 100 µL of factor Xa-Va in the presence of calcium and 50 µL prothrombin were added, followed by a 10-min incubation at 37 °C. After five washing steps with 300 µL of washing solution, 50 µL of a chromogenic substrate was added. After formation of a chromogenic substance after 3 min of incubation at 37 °C, 50 µL of 2% citric acid was added to stop the reaction, and the adsorption was measured at 405 nm.

**3.7 *In-vitro* evaluation** $\boldsymbol{\beta}$**-III tubulin differentiation markers labelling on the SH-SY5Y**

In short, the medium was removed, and then cells were rinsed with PBS and incubated with 2% paraformaldehyde for 30 min at room temperature for fixation. Cells were next permeabilized with 0.2% PBS-Triton X-100 for 20 min at room temperature, and non-specific binding was blocked by 1% BSA in PBS for 1 h. Cells were incubated with primary anti-β-III tubulin overnight at 4 °C. The next day, wells were washed with PBS and an Alexa Fluor-488-conjugated goat anti-rabbit immunoglobulin G (IgG) antibody (Abcam) was added, and after 1 h, DAPI was used to label nuclei. Immunofluorescence images were observed with a Leica DMi8 fluorescence microscope (Sage Vision, West Chester, PA, USA). Three random images were taken per treatment in three independent experiments, and the fluorescence intensity of β-III tubulin was quantified by the integrated fluorescence density using ImageJ software (1.6, NIH, Bethesda, MA, USA).

**3.7.2 LUHMES cells differentiation**

LUHMES cells (at 2×10^6^) were seeded in Advanced DMEM/F12 culture medium containing N-2 supplement and 2 mM L-glutamine (ThermoFisher Scientific), and 40 ng/mL recombinant basic fibroblast growth factor (bFGF; R&D Systems) in a flask pre-coated with 50 µg/mL poly L-ornithine (Sigma-Aldrich, St. Louis, MO, USA) and 1 µg/mL fibronectin (Sigma-Aldrich). Cells were left to proliferate in a 5% CO_2_, 37 °C humidified incubator. For differentiation, 2.5×10^6^ LUHMES cells were seeded in a 75-mL flask and incubated in proliferation medium. After 24 h, the medium was replaced with Advanced DMEM/F12 (ThermoFisher) supplemented with N-2 supplement, 2 mM L-glutamine, 1 mM dibutyryl cAMP (Sigma-Aldrich), 1 μg/mL tetracycline (Sigma-Aldrich) and 2 ng/mL recombinant human BDNF (R&D Systems) and allowed to differentiate for 2 days. Cells were next transferred into 24-well plates to complete the differentiation process for an additional 3 days as described before [1].

**3.8 Assessment of functionality in a TBI *mice model***

**3.8.1 *CCI***

A mild TBI was induced in mice, using a eCCI 6.3 (Custom Design & Fabrication, Sandston, VA, USA) essentially as we described before [3]. Briefly, after complete anesthetization with a mixture of zoletil and xylazine, the head of a mouse was immobilized in a stereotaxic frame using ear bars. A midline incision was created to expose the skull, and then a hole 4 mm in diameter was carefully cut over the right hemisphere between the bregma and lambda. A mild injury was next induced using an impactor with 3-mm tip at a velocity of the actuator of 3 m/s, a deformation depth of 0.2 mm, and a dwell time of 250 ms. The injury was initiated by hitting the surface of the cortex perpendicularly. Then, the skin was sutured, and antibiotic ointment was applied afterwards to prevent infection. Mice were placed in a heated cage for recovery.

**3.8.2 Gene expression analysis by qPCR of the injury cortex**

Total RNA was purified from collected mice cortices (*n*=5~7/group) using a RNeasy Lipid Tissue Mini Kit 50 (cat. no. 74804, Qiagen, Germantown, MD, USA), according to the manufacturer’s recommendations. The total RNA concentration was quantified using a microplate spectrophotometer (Take3 BioTEx, Winooski, VT, USA). RNA (1 μg) was used to synthesize complementary (c)DNA using the Applied Biosystems High-Capacity cDNA reverse-transcription kit (no. 4368814, Waltham, MA, USA). Retro-transcription was carried out using the following program on a StepOneTM Real-Time PCR System (ThermoFisher Scientific): 10 min at 25 °C, 60 min at 37 °C, 60 min at 37 °C, 5 min at 85 °C, and 5 min at 4 °C. cDNA was stored at -20 °C prior to performing the qPCR analysis. Power SYBR Green PCR Master Mix (5 μL, cat. no. 4367659, ThermoFisher Scientific), 0.1 μL of the forward primer, 0.1 μL of the reverse primer, 2 μL of cDNA pre-diluted 20 times, and 2.8 μL of RNase-free water were included in each sample. The StepOne^TM^ Real-Time PCR System was used with the following amplification profile: 50 °C for 12 min and 95 °C for 10 min, followed by 40 cycles of 95 °C for 15 s, 60 °C for 30 s, and 95 °C for 15 s, with 60 °C for 1 min as a step-and-hold melt curve analysis. Several inflammatory gene expressions related to astrocytes, microglia, and cytokine activation were quantified, and primers used are described in Supplementary Table 1.

**3.9 References**

[1] Chou ML, Wu JW, Gouel F, Jonneaux A, Timmerman K, Renn TY, et al. Tailor-made purified human platelet lysate concentrated in neurotrophins for treatment of Parkinson's disease. Biomaterials. 2017;142:77-89.

[2] Nebie O, Barro L, Wu YW, Knutson F, Buee L, Devos D, et al. Heat-treated human platelet pellet lysate modulates microglia activation, favors wound healing and promotes neuronal differentiation in vitro. Platelets. 2021;32:226-37.

[3] Nebie O, Carvalho K, Barro L, Delila L, Faivre E, Renn TY, et al. Human platelet lysate biotherapy for traumatic brain injury: preclinical assessment. Brain. 2021;144:3142-58.

[4] Barro L, Delila L, Nebie O, Wu Y, Knutson F, Watanabe N, et al. Removal of minute virus of mice-mock virus particles by nanofiltration of culture growth media supplemented with 10% human platelet lysate. Cytotherapy. 2021;23:S176.

[5] Barro L, Su YT, Nebie O, Wu YW, Huang YH, Koh MB, et al. A double-virally-inactivated (Intercept-solvent/detergent) human platelet lysate for in vitro expansion of human mesenchymal stromal cells. Transfusion. 2019;59:2061-73.

[6] Burnouf T, Tseng YH, Kuo YP, Su CYJT. Solvent/detergent treatment of platelet concentrates enhances the release of growth factors. 2008;48:1090-8.

[7] Chou ML, Lin LT, Devos D, Burnouf T. Nanofiltration to remove microparticles and decrease the thrombogenicity of plasma: in vitro feasibility assessment. Transfusion. 2015;55:2433-44.

[8] Delila L, Wu YW, Nebie O, Widyaningrum R, Chou ML, Devos D, et al. Extensive characterization of the composition and functional activities of five preparations of human platelet lysates for dedicated clinical uses. Platelets. 2021;32:259-72.
